# Sex specific trajectories of central adiposity, lipid indices, and glucose level with incident hypertension: 12 years Follow-up in Tehran lipid and glucose study

**DOI:** 10.1186/s12967-021-02749-x

**Published:** 2021-02-23

**Authors:** Noushin Sadat Ahanchi, Seyed Saeed Tamehri Zadeh, Davood Khalili, Fereidoun Azizi, Farzad Hadaegh

**Affiliations:** 1 Prevention of Metabolic Disorders Research Center, Research Institute for Endocrine Sciences, Shahid Beheshti University of Medical Sciences, No. 24, Parvaneh Street, Velenjak, Tehran, Iran; 2 School of Medicine, Tehran University of Medical Sciences, Tehran, Iran; 3 Department of Biostatistics and Epidemiology, Research Institute for Endocrine Sciences, Shahid Beheshti University of Medical Sciences, Tehran, Iran; 4 Endocrine Research Center, Research Institute for Endocrine Sciences, Shahid Beheshti University of Medical Sciences, Tehran, Iran

**Keywords:** Waist circumference, Triglyceride, Trajectory, Hypertension

## Abstract

**Aims:**

To identify sex specific trajectories of waist circumference (WC),triglyceride (TG), total cholesterol (TC), high density lipoprotein cholesterol (HDL-C) and fasting plasma glucose (FPG) during adulthood and examine their associations with incident hypertension.

**Methods:**

The cohort consisted of 5030 participants (2051 males) with at least 2 repeated measurement during a median of 12 years follow up. We identified trajectory groups using latent class growth mixture model, their association with hypertension was examined using multivariate Cox-regression analysis.

**Results:**

We found 997 cases of hypertension (483 male). For both exposures, three distinct trajectory groups were identified in both genders. For WC, in women: low-increasing, 82.4%; high-stable, 13.4%; high-increasing, 4.2% and in men: stable, 94.6%; low-increasing, 3.6% and for high- increasing, 1.7%. For TG, in women: stable, 91.3%; decreasing, 5.9%; inverse U-shape, 2.8%; in men: stable, 89.7%; inverse U- shape, 6.2% and for decreasing, 4.1%.

Regarding WC, high stable and high-increasing trajectories were associated with hypertension in the multivariate model [(hazard ratio (HR) = 1.66 (95% CI 1.26–2.20) and 2.78(1.79–3.60), respectively]. Among men, this association was shown only for the low-increasing trajectory [2.76: 1.49–5.10]. For TG, among women decreasing and inverse U-shape trajectories were significantly associated with hypertension in the multivariate model [1.32:1.01–1.76] and [2.23:1.58–3.23, respectively].

We did not find any impact of increasing trajectories of FPG and HDL-C on incident hypertension. Considering TC, all individuals followed a stable trajectory.

**Conclusion:**

WC dynamic changes in both gender and TG trajectory among women were significantly associated with incident hypertension.

## Introduction

Based on many years of experience, hypertension significantly increases individuals’ vulnerability to cardiovascular disease (CVD) and mortality. Despite tremendous advancements regarding hypertension prevention and treatment, itremainsa global concern, especially in the Middle East and North Africa (MENA) region [1, 2]. Apart from CVD, hypertension is a major factor in the development of end-stage renal disease, heart failure, and premature mortality [3]. Approximately 40% of the people of the Eastern Mediterranean region (EMR) suffer from hypertension, which is similar to the prevalence of global hypertension among adults who are older than 25 [4].

Lines of research have been performed so as to identify the possible risk factors implicated in hypertension development [5, 6]. An updated systemic review has addressed the pivotal role of several factors including, but not limited to, age, sex, body mass index (BMI), waist circumference (WC), lipid abnormalities, smoking and diabetes in hypertension [6]. It is of importance to note that the majority of studies assessing the association between potential risk factors and incident hypertension have focused on a snapshot measurement of those and ignored the impact of their dynamic changes on incident hypertension.

Although the association between general adiposity and incident hypertension has been investigated in several research [7, 8], to the best of our knowledge, so far there is no information available on the effects of central adiposity, lipid indices including triglyceride (TG), total cholesterol (TC) and high-density lipoprotein cholesterol (HDL-C), and glucose level trajectories over the course of a life-time on hypertension development. Therefore, we examined the sex specific trajectories of central adiposity, lipid indices, and glucose level for incident hypertension, using Latent Class Growth Mixture Modeling (LCGMM), in the oldest cohort of the MENA region, namely the Tehran Lipid and Glucose study during a follow-up of more than 12 years.

## Methods

### Participants

Tehran Lipid and Glucose Study (TLGS) is a dynamic prospective population-based study conducted on a representative sample of Tehranians recruited from among residents of district 13 of Tehran. The main groups of ethnicities in Iran are the Caucasians with the most prevalence (75.4%) and the Turks (22%). The distribution of ethnicity of Tehran is virtually similar to distribution of ethnicity of Iran [9]. It aims to determine the prevalence and incidence of non-communicable disease risk factors and to develop and implement a healthy lifestyle. The age distribution of the TLGS population at baseline is representative of the overall population of Tehran (Iran National Census, 1996). Details of study design, sampling and rationale hasbeen published elsewhere [10]. In brief, TLGS enrollment was carried out in two phases, the first of which was from January 31, 1999 to July 03, 2001, with a second enrollment phase from October 20, 2001 till September 22, 2005. Data collection is planned to continue for at least 20 years at approximately 3-year intervals (i.e. phase III: 2005–2008, phase IV: 2008–2011, phase V: 2011–2014, and phase VI: 2015–2018). For the current study, we requited 6750 participants aged ≥ 18 and   ≤ 40 years (5294 participants from phase 1 and 1456 participants from phase 2). The mean baseline age was 28.87 years (18 to 40 years at baseline and 20 to 60 years at follow-up). Exclusion criteria included prevalent hypertension (n = 1056) and missing data on BP measurements, WC, TG, HDL-C, TG and FPG (n = 300, considering overlap features between numbers), covariates including BMI, smoking and diabetes status (n = 56), and finally those without any follow-ups after enrolment (n = 308) leading to a total of 5030 (2051 of which were male) for data analysis (Additional file 1: Figure S1). Moreover, Based on TLGS protocol waist circumference is not measured for pregnant women. In the current studyWCfor pregnant women were not considered in the analysis if they were pregnant for those relevant phases (i.e., 52, 54, 28, 18 and 8 pregnant women in the follow up 1, 2, 3, 4, and 5 respectively). WC and TG, HDL-C, TG, FPG data after the onset of hypertension (outcome) were not included in the analyses.

## Clinical and laboratory measurements

Using a standard questionnaire, a trained interviewer collected information including demographic data, drug history and past medical history of diabetes mellitus. Details of anthropometric measurements including weight and height have been reported elsewhere [10]. Weight was measured using digital scale and was recorded to the nearest 100 g, without shoes and with light clothing. Height was measured in a standing position, without shoes. BMIwas calculated by dividing weight (kg) by square of height (m2). WC was measured with light clothing at the level of the umbilicus, excluding for pregnant women at each phase. After a 15-min rest in the sitting position, two measurements of systolic blood pressure (SBP) and diastolic blood pressure (DBP) were measured by trained personnel, on the right arm, using a standardized mercury sphygmomanometer (calibrated by the Iranian Institute of Standards and Industrial Researches); the mean of the two measurements was considered as the participant’s BP. A blood sample was taken between 7:00 and 9:00 AM for all study participants, after 12 to 14 h of overnight fasting. Levels of fasting blood glucose (FBS), total and TG were enzymatically measured [10].

### Definition of terms

Hypertension was defined as SBP ≥ 140 mmHg or DBP ≥ 90 mmHg or the use ofanti-hypertension medications. BMI was calculated as weight (kg)/height (m2), smoking status was divided into three categories (current/past/never), A current smoker was defined asa person who smokes cigarettes or uses other tobacco products daily or occasionally. A past-smoker was a formerly daily or occasional smoker who currently does not smoke and non-smokers were defined as people who had never smoked. Diabetes mellitus was defined as FPG ≥ 7 mmol/L or use of anti-diabetic medications.

### Statistical analyses

Statistical analyses were conducted using the Mplus7.4 (Muthén and Muthén [12]), the SPSS 17.0 and Stata14 software packages. The analyses for the current study consisted of two steps, described in detail below.

### Step 1. Latent class growth (mixture) modeling

Heterogeneity in longitudinal development of WC, TG, HDL-C, TC and FPG was analyzed using Latent Class Growth (Mixture) Modeling (LCGMM) [11, 12]. LCGMM is a contemporaneous longitudinal method based on structural equation modeling, including both continuous and categorical latent variables. The method is an extension of usual growth modeling. In conventional analyses, the theory that all individuals in the study sample come from a single population should hold; meaning that one (average) trajectory will adequately address the developmental pattern of the sample. This hypothesis is relaxed in LCGMM, meaning that individuals in the sample need not come from one single underlying population, but can come from multiple latent sub-clusters.

Finding the number and characteristics of these underlying sub-clusters of individuals is the main goal of LCGMM. This is done by identifying k number of distinguished latent classes of, in the present study, trajectories of WC, TC, HDL-C, FPG and TG. Each identified class has its own particular growth parameters (intercept, slope), which are also presupposed to be unobserved, or latent. In addition, LCGMM can also account for within-class variation in either, or both, of the growth parameters, implying within-class heterogeneity on top of the between-class heterogeneity in initial status (intercept) or growth (slope). Varied LCGMM models were run before we chose a final model [13]. First, various linear LCGMM with fixed intercept and slope variance within classes were assessed. Then, quadratic slopes were added to the model permitting for curved developmental patterns. To understand the possibility of different trajectories by gender, these steps were run separately among men and women. Mplus did not compute the standard error in our figures.

### Model selection (class identification)

To identify the optimal number of classes, a common forward procedure was done, starting with a one class solution (i.e., assuming all individuals follow a similar trajectory over time), next adding more classes one at time to examine whether or not the model fit improves due to the additional class. The number of groups were determined by the following criteria: Bayesian information criteria (BIC), entropy, Akaike information criteria (AIC), Adjusted Lo-Mendell-Rubin likelihood ratio test (A LMR test), class size and interpretability [14]. BIC has been shown to be a robust indicator of the preferred k class solution. Lower BIC values indicate better model fit [15]. The AIC evaluates a model or partition scheme by combining the maximum log-likelihood value with a penalty that depends on the number of parameters being estimated. Models that generate the lowest values are optimal [15]. Entropy is an index for assessing the precision of assigning latent class membership. Higher entropy values indicate greater precision of classification [16]. A LMR test assesses the differences in the log-likelihood between LCGA models with k and k + 1 clusters, where p-values < 0.05 indicates a benefit of adding an additional cluster [17]. We also took the posterior probabilities to assign subjects to the trajectories. Each individual in the sample we reassigned to their most-likely class based on these probabilities which is the highest probability. Therefore, the classes are clearly distinguished from each other. Based on posterior probability, persons were assigned to the trajectory that best matched their work engagement; a probability > 0.8 is recommended and a probability closer to 1 indicates better classification [14]. For each individual in the sample, the probability of belonging to each of the k classes is estimated. The probability for the class to which the individual was assigned, should be considerably higher than the other probabilities. By this means, the classes are clearly distinguished from each other. Clinical interpretation and class sample size were also considered in the decision-making process. We rejected models with clinically uninterpretable classes and classes with < 1% of the total sample. Finally, we considered the usefulness of the separate classes in practice. LCGMM models are computationally-heavy models, often with convergence issues or hitting local maxima. Mplus incorporates random starts to avoid these issues as much as possible. Missing data were adequately handled by the Expectation–Maximization algorithm, which is an iterative method to find maximum likelihood estimation with robust standard errors by missing at random (MAR) assumption.

### Step 2. Association between the trajectory group membership and incident hypertension

The first step of the analyses provided us with a classification of the study sample into distinct trajectory subgroups, coded as a categorical variable with k number of categories. Sex stratify Cox proportional hazard models with follow-up time as the time scale were used to examine the association between trajectory classes of TG, WC, FPG, HDL-C and incident hypertension, considering the trajectory with highest prevalence as reference. We considered multiple potential confounders based on our previous study, and then we included age, SBP, DBP, body mass index (BMI), diabetes, HDL-C, ascovariate. All other statistical tests were two-sided and statistical significance was determined as p < 0.05.

## Results

Baseline and end point characteristics are shown in Table 1.

**Table 1:** characteristics of study population at the baseline and end point*

	Baseline	End point	P Value
Females (n = 2979)			
Age (years)	28.63 (6.60)	42.12 (7.55)	0.00
Smoking status			
(Current) (%)	0.6	2.3	0.06
(paste) (%)	2.3	4.1	
BMI (kg/m2)	25.24 (4.66)	28.45 (5.10)	0.00
WC (cm)	80.46 (11.32)	91.34 (11.62)	0.02
FPG (mg/dL)	87.26 (14.43)	94.01 (21.06)	0.01
TG (mg/dL)	99 (72–143)	111 (79–157)	0.04
HDL_C	44.60 (10.30)	50.20 (11.47)	0.02
TC	180 (157–206)	186 (164–212)	0.05
Males (n = 2051)			
Age (years)	29.22 (6.67)	42.33 (7.58)	0.00
Smoking status			
(Current) (%)	26.4	29.2	0.06
(Paste) (%)	9.2	13.1	
BMI (kg/m2)	24.87 (4.21)	27.65 (4.18)	0.03
WC (cm)	85.14 (11.44)	96.48 (10.91)	0.03
FPG (mg/ dL)	88 (83–94)	96 (87–101)	0.04
TG (mg/dL)	133 (91–198)	147 (103–206)	0.03
HDL-C	37.88 (9.05)	41.39 (9.84)	0.03
TC	182 (159–211)	188 (164–215)	0.05

Data are mean (standard deviation) for continues, and frequency (%) for categorical variables *incident hypertension/or the last follow-up

*BMI* Body mass index, *DBP* Diastolic blood pressure, *SBP* Systolic blood pressure, *TG* Triglyceride, *TC* total cholesterol, *FPG* fasting plasma glucose

### WC, TG, HDL-C, FPG and TC trajectories for Incident BP

The study population included 2051 males and 2979 females with mean (SD) age of 29.2(6.6) and 33.9(8.4) years, respectively. During follow-up, we found 997 cases (males = 483) with incident hypertension.

#### WC trajectories 

**Fig. 1:**
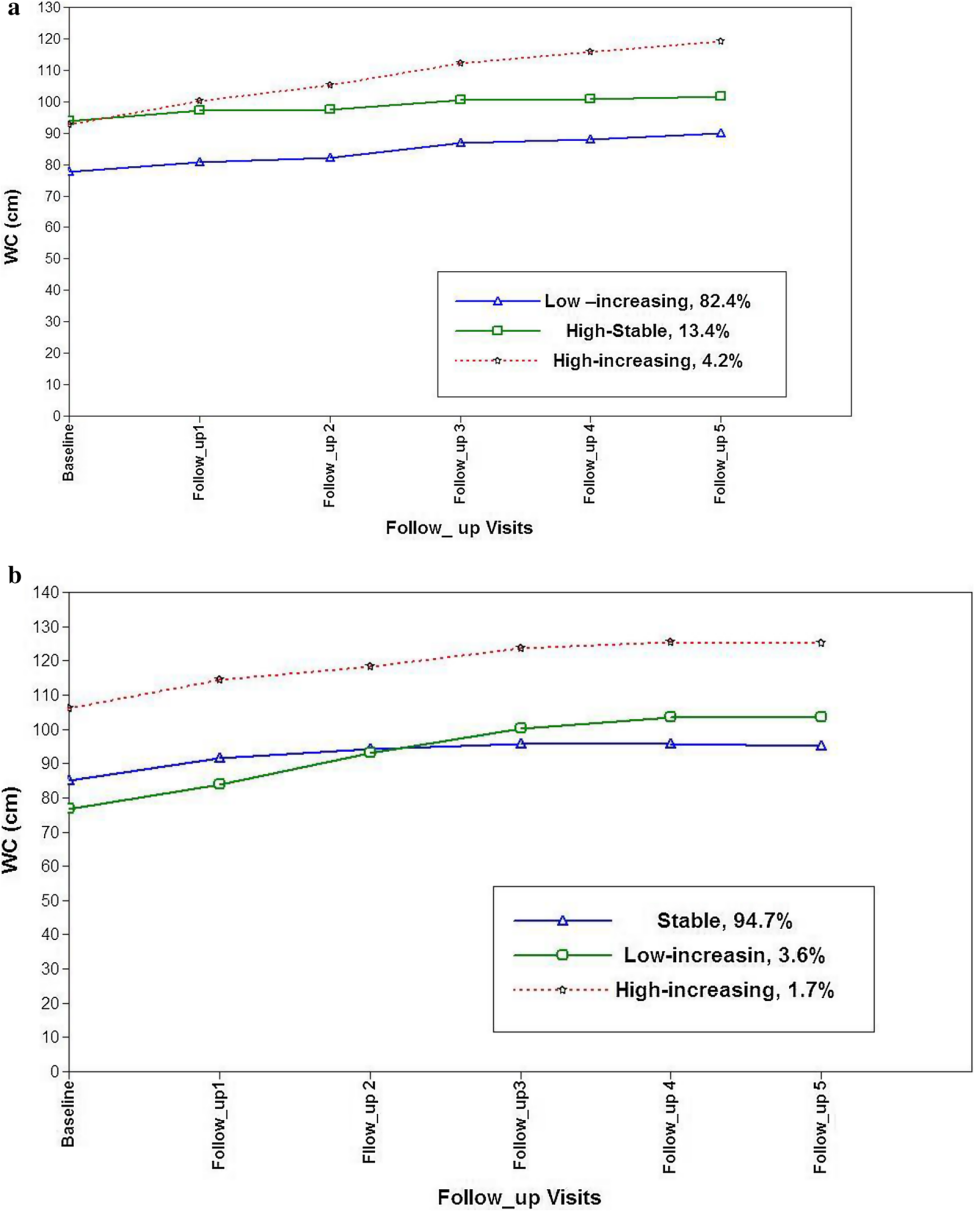
Linear latent trajectory classes of waist circumference among women for incident hypertension.** b** latent trajectory classes of waist circumference among men for incident hypertension

We performed LCGMM analysis to explore both linear and quadratic terms to fit the model. Model selection between linear and quadratic term was done using CFI, SRMR and TLI; only linear models were applied. Additional file 2: Table S1 shows the model fit statistics for the growth mixture model with 2 to 6 classes. In both genders, the 4- and 5-class models resulted in some classes that included less than 1% of the sample, hence, these models were excluded from further consideration. Therefore, the 3-class model was chosen as the optimal class size because it had the lowest BIC, sample size adjusted BIC and AIC among the remaining models. For females, three waist trajectory classes can be described as: Trajectory 1(low–increasing, n = 2606, 82.4%), Trajectory 2 (High-stable, n = 275, 13.4%) and Trajectory 3(high-increasing, n = 984.2%).For males, the three waist trajectory classes can be described as: Trajectory 1(stable, n = 1987, 94.6%), Trajectory 2 (low-increasing, n = 43, 3.6%); and Trajectory 3(high-increasing = 21, 1.7%) (Fig. 1). Baseline characteristics of the study population by WC trajectory classes are shown in Table 2. Accordingly, among females, participants included in the low–increasing Trajectory (reference group), generally had more favorable baseline characteristics than High-stable and high- increasing rajectories (i.e. lower age, WC, TG, SBP and DBP). Among males, participants in the stable trajectory (reference group) were older and had the lowest incidence of hypertension. However, in women, the lowest incidence of hypertension was found among participants in the low–increasing trajectory.

**Table 2:** Baseline characteristics of study population by waist circumference trajectories for incident hypertension

Females	Low–increasing (n = 2606)	High-Stable (n = 275)	High-increasing (n = 98)	P value
Age (years)	28.15 (6.60)	32.78 (5.17)	29.52 (5.93)	0.000
Smoking status				0.04
Current) (%)	0.7	0.4	0.0	
(Paste) (%)	2.1	4.7	4.1	
BMI (kg/m2)	24.21 (3.77)	32.56 (3.4)	32. 01 (5.25)	0.000
WC (cm)	77.93 (9.17)	95.00 (7.13)	95.04 (8.01)	0.000
Diabetes, n (%)	20 (0.8)	12 (4.4)	0	0.000
TG (mg/dL)	95 (70–133)	148 (109–206)	125 (83–174)	0.000
SBP (mmHg)	106.7 (10.26)	111.28 (9.70)	111.08 (9.53)	0.000
DBP (mmHg)	71.91 (8.12)	76.1 (7.18)	75.77 (6.94)	0.000
Hypertension, n (%)	362 (13.9)	104 (37.8)	48 (49)	0.000

Males	Low- increasing (n = 43)	Stable (n = 1987)	High-increasing (n = 21)	P value
Age (years)	22.16 (5.39)	29.40 (6.61)	26.95(6.32)	0.000
Smoking status				0.2
(Current) (%)	18.6	26.4	14.3	
(Paste) (%)	4.7	9.8	4.8	
BMI (kg/m2)	20.82 (2.42)	24.85 (4.05)	35.4 (4.67)	0.000
WC (cm)	73.72 (6.30)	85.11 (11.07)	111.42 (12.56)	0.000
Diabetes, n (%)	0	28 (1.4)	0	0.04
TG (mg/dL)	89 (70–113)	135 (91–200)	164 (122–217)	0.000
SBP (mmHg)	105.48 (11.72)	112.39 (10.42)	121.76 (9.74)	0.000
DBP (mmHg)	70.46 (9.50)	73.77 (8.13)	77.47 (9.88)	0.000
Hypertension, n (%)	12 (27.9)	460 (23.2)	11 (52.4)	0.006

Data are mean (standard deviation) for continuous, and frequency (%) for categorical variables. *BMI* Body mass index, *DBP* Diastolic blood pressure, *SBP* Systolic blood pressure, *TG* Triglyceride,* WC* Waist circumference

As shown in Table 3, among females, compared to the reference group, high stable and high-increasing trajectories were significantly associated with hypertension in the fully adjusted model [HR = 1.66 (95% confidence interval (CI): ((1.26–2.20))], [HR = 2.78 (95% confidence interval (CI): (1.79–3.60))]. In women, when we replaced the high-stable with thelow- increasing trajectory as a reference group, the high- increasing trajectory showed a significant risk even in the fully adjusted model (1.49 (1.06–2.11)).

**Table 3:** Associations between waist circumference trajectories and hypertension

	Model1			Model2			Model3		
	Adjusted HR (95% CI)	β	P value	Adjusted HR (95% CI)	β	P value	Adjusted HR (95% CI)	β	P value
Females									
Low-increasing	Reference			Reference			Reference		
High-stable	2.21 (1.76–2.76)	.79	0.000	1.68 (1.27–2.21)	.51	0.000	1.67 (1.26–2.20)	0.50	0.000
High- increasing	3.28 (2.43–4.45)	1.19	0.000	2.50 (1.76–3.50)	.91	0.000	2.78 (1.79–3.60)	0. 93	0.000
Males									
Stable	Reference			Reference			Reference		
Low–increasing	2.47 (1.38–4.43)	0.90	0.002	2.85 (1.58–5.13)	1.04	0.002	2.76 (1.49–5.10)	1.01	0.001
High-increasing	2.68 (1.46–4.90)	0.98	0.001	1.48 (0.76–2.89)	0.39	0.24	1.44 (0.74–2.81)	0.36	0.28

*CI* Confidence interval, *HR* Hazard ratio

Model1 was adjusted for age, SBP, DBP

Model2 was adjusted for age, SBP, DBP, BMI

Model3: was adjusted for age, SBP, DBP, Smoking Statu, Diabetes, BMI, HDL-C

As shown in Table 3, among males, the low-increasing trajectory was significantly associated with hypertension in the fully adjusted model [HR = 2.76 (95% confidence interval (CI): (1.49–5.10)]. However, the significant association between high-increasing and incident hypertension 2.68 (1.46–4.90) significantly attenuated after adjustment for BMI (Model 2). In men, when we replaced the High-increasing group by the stable group as the reference, the Low–increasing trajectory show higher, but not significant risk in none of the models (Additional file 2: Table S8).

#### TG Trajectories

**Fig. 2:**
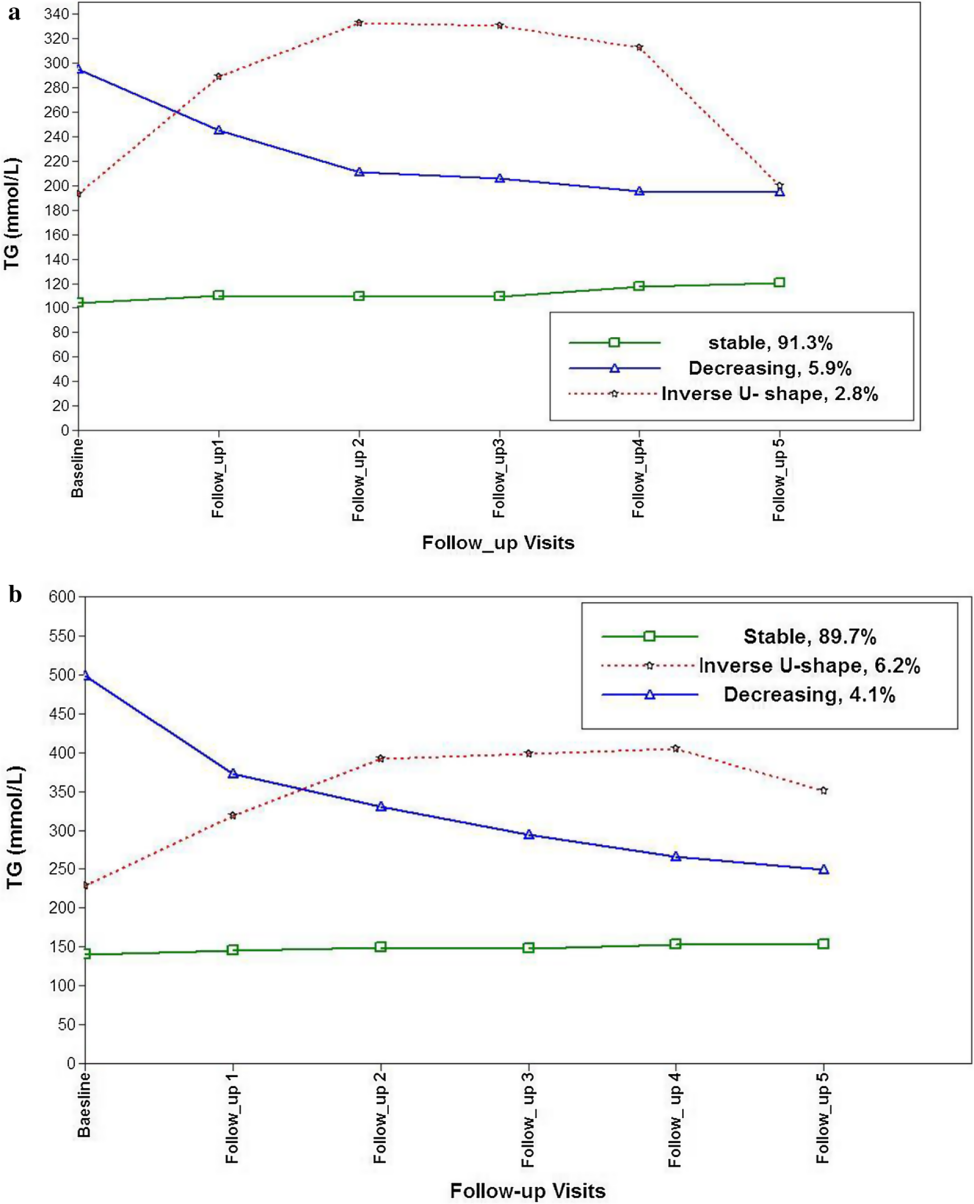
**a** Quadratic latent trajectory classes of triglycerides among women for incident hypertension. **b** Quadratic latent trajectory classes of triglycerides among men for incident hypertension

Results of LCGMM analysis showed that the quadratic terms function was the best model. Table 4 shows the quadratic model fit statistics for 2 to 6 classes. As showed in the table, the 4- and 5-class models included less than 1% of participates and hence were excluded from further consideration. In both genders the 3-class model, was chosen as the optimal class due to the lowest BIC, sample size adjusted BIC and AIC between the remaining models (Additional file 2: Table S3). In females, Trajectory 1(stable**,** n = 2740, 91.3%), Trajectory 2(decreasing, n = 158, 5.9%) and Trajectory 3 (inverse U-shape, n = 81, 2.8%) were found. In males Trajectory 1(stable, n = 2740, 89.7%), Trajectory 2(inverse U-shape, n = 121, 6.2%), Trajectory 3(decreasing, n = 85, 4.2%) were identified (Fig. 2). Baseline characteristics of the study population by waist trajectory classes are shown in Table 4. Accordingly, among females, participants included in the stable trajectory generally had more favorable baseline characteristics than inverse U-shape and decreasing trajectories (i.e. lower age, WC, FPG, TG, SBP and DBP). Among men (similar to women) participants in the stable trajectory had more favorable baseline characteristics than other trajectories, excluding participants’ age which was lower in the inverse U-shape trajectory. In both genders, the lowest incidence of hypertension was found among participants in the stable trajectory.

**Table 4:** Baseline characteristics of study population by triglyceride trajectories for incident hypertension

Females	Stable (n = 2740)	Inverse U-shape (n = 81)	Decreasing (n = 158)	P value
Age (years)	28.26 (6.57)	29.52 (5.93)	32.78 (5.77)	0.000
Smoking status				
(Current) (%)	0.5	2.9	0.6	
(Paste) (%)	2.2	3.7	3.8	
BMI (kg/m2)	24.98 (4.59)	26.99 (4.32)	28.80 (4.22)	
WC (cm)	79.73 (11.06)	86.85 (10.73)	90.52 (10.13)	0.000
Diabetes n (%)	18 (0.7)	6 (7.4)	8 (5.1)	0.000
TG ( mg/dL)	95 (70–133)	128 (168–233)	290 (260–324)	0.000
SBP (mmHg)	106.96 (10.17)	108.08 (10.53)	113.25 (10.30)	0.000
DBP (mmHg)	72.07 (8.09)	74.67 (7.54)	77.16 (6.88)	0.000
BP, n (%)	424 (15.5)	32 (39)	58 (36.7)	0.000

Males	Stable (n = 1845)	Inverse U-shape (n = 121)	Decreasing (n = 85)	P value
Age (years)	29.04 (6.76)	28.70 (6.34)	33.89 (4.39)	0.000
Smoking status				
(Current) (%)	25.4	32.2	32.9	
(Paste) (%)	9.3	8.3	9.4	
BMI (kg/m2)	24.68 (4.17)	25.3 (4.13)	28.27 (3.51)	0.01
WC (cm)	84.61 (11.36)	85.89 (11.33)	94.75 (9.11)	0.000
Diabetes, n (%)	28 (1.1)	2 (1.7)	6 (7.1)	0.04
TG ( mg/dL)	124 (87–181)	215 (144–288)	467 (409–571)	0.000
SBP (mmHg)	112.06 (10.48)	113.43 (11.6)	116.92 (9.52)	0.000
DBP (mmHg)	73.52 (8.14)	73.55 (8.1)	78.67 (8.12)	0.000
BP, n (%)	416 (22.5)	32 (26.4)	35 (41.2)	0.000

Data are mean (standard deviation) for continues, and frequency (%) for categorical variables

*BMI* Body mass index, *DBP* Diastolic blood pressure, *SBP* Systolic blood pressure; *TG* Triglyceride

As shown in Table 5, among females, compared to the stable trajectory as a reference group, the inverse U-shape and decreasing trajectories were significantly associated with hypertension in the fully adjusted model [HR = 1.36 (95% confidence interval (CI): ((1.02–1.80))], [HR = 2.25 (95% confidence interval (CI): (1.58–3.27)),]. When we replaced the decreasing trajectory with the stable trajectory as a reference group, the inverse U-shape trajectory showed a significant risk even in the fully adjusted model (1.64 (1.05–2.54)).As shown in Table 5, among males, a 43% increased risk of hypertension in the decreasing trajectory (1.01–2.04) significantly attenuated after adjusting for BMI (Model 2). In men when we replaced the High- increasing group by the stable group as the reference, the Low –increasing trajectory did not show higher significant risk in none of the models.

**Table 5:** Associations between Triglyceride trajectories and Hypertension

	Model1			Model2			Model3		
	Adjusted HR (95% CI)	β	P value	Adjusted HR (95% CI)	β	P value	Adjusted HR (95% CI)	β	P value
Females									
Stable	Reference			Reference			Reference		
Inverse U-shape	2.45 (1.70–3.51)	0.89	0.000	2.27 (1.57–3.26)	0.82	0.000	2.23 (1.58–3.23)	0.80	0.000
Decreasing	1.45 (1.09–1.93)	0.37	0.009	1.36 (1.02–1.80)	0.03	0.031	1.32 (1.01–1.76)	0.30	0.033
Males									
Stable	Reference			Reference			Reference		
Inverse U shape	1.07 (0.74–1.52)	0.07	0.700	1.05 (0.73–1.50)	0.28	0. 77	1.09 (0.75–1.55)	0.09	0.69
Decreasing	1.43 (1.01–2.04)	0.36	0.042	1.33 (0.93–1.88)	0.52	0. 11	1.36 (0.95–1.89)	0.30	0.08

*CI* Confidence interval, *HR* Hazard ratio

Model 1was adjusted for age, SBP, DBP

Model 2was adjusted for age, SBP, DBP, BMI

Model 3: was adjusted for age, SBP, DBP, Smoking Status, Diabetes, BMI, HDL-C

#### HDL-C, FPG and TC trajectories

We performed LCGMM analysis to explore both linear and quadratic terms to fit the model. Model selection between linear and quadratic term was done using CFI, SRMR and TLI; only linear models were applied. For HDL-C, FPG. In both genders, the 2-class model was chosen as the optimal class size because it had the lowest BIC, sample size adjusted BIC and AIC among the remaining models. For TC in both gender all individuals followed a similar trajectory and 1-class model was chosen as the optimal class. For females, tow FPG trajectory classes can be described as: Trajectory 1(Stable, n = 2927, 98.3%), Trajectory 2 (increasing, n = 52, 1.7%) and tow HDL-C trajectory classes can be described as: Trajectory 1(Low increasing, n = 2759, 89.6%), Trajectory 2 (High increasing, n = 220, 10.4%) one TC trajectory can be described as Trajectory 1(Stable, n = 2979, 100%).

For males, the tow FPG trajectory classes can be described as: Trajectory 1(stable, n = 1980, 96.5%), Trajectory 2 (increasing, n = 71, 3.6%), tow HDL-C trajectory classes can be described as: Trajectory 1(Low increasing, n = 1913, 91.6%), Trajectory 2 (High increasing, n = 131, 8.4%) one TC trajectory can be described as Trajectory 1(Stable, n = 2051, 100%) Additional file 3, Additional file 4, Additional file 5. Baseline characteristics of the study population by FPG, HDL-C and TC trajectory classes are shown in Additional file 2: Tables S3, S5 and S7. As shown in Additional file 2: Tables S4, S6, we did not find any impact of increasing level of FPG and HDL-C on incident hypertension compared with their reference groups. Considering TC, all individuals followed a similar trajectory.

## Discussion

To our knowledge, this is the first population-based study that examined the sex-specific trajectories of central adiposity (as assessed by WC), dyslipidemia (as assessed by TG, HDL-C, and TC level), and FPG for incident hypertension among the middle-aged adult population, during more than a decade offollow-up. Using the LCGM method, 3 distinct groups of WC and TG, 2 distinct groups of HDL-C and FPG, and only one group of TC changes were found in each gender. In addition, we examined the associations of these trajectory groups with incident hypertension in different models.

## WC trajectory and incident hypertension

For WC in our study, among women in the high-stable group (consisting of about 13% of the total population) waist circumference stayed stable at around 95 cm, while the WC of the other two groups (about 87% of total population) increased with different baseline levels and incremental slopes. Interestingly, among women, the majority of our cohort (low-increasing trajectory, reference group) showed the increasing value of WC, but incident hypertension was low in that group; the issue might be attributable to that this value did not capture the threshold of 90 cm, the cut-off point for the development of hypertension in both genders among Iranian population [18]. The increasing trend of high WC might be attributed to progressive decrease in physical activity level among Iranian women mainly related to decrease in work-related activity [19]. We found that female participants in the high-increasing group showed a more than 2.5-fold increased risk of hypertension compared to the reference group, after adjustment for age, BMI, SBP, DBP, smoking status, diabetes and HDL-C. Although participants in the high-stable and high-increasing groups had the same intercept for WC, but when considering the high stable group as reference, participants in the high-increasing group showed a 50% higher risk of hypertension.

Among men, WC trajectories had a steeper initial WC increase in about 5% of the population, however, the majority had stable WC levels over time. Participants in the low- increasing group (i.e. WC 78 at the onset and 103 cm at last follow-up) showed an approximately 2.5-fold increased risk of hypertension compared to those in the stable group. However, for the high- increasing group with a mean BMI of around 35 kg/m^2^ and WC of 107 at onset, this risk was not independent of baseline BMI; this indicates that increasing WC does not have significant role in predicting hypertension in individuals with moderate level of obesity at baseline. Actually, general adiposity remained as an independent risk factor in the model [hazard ratio of BMI = 1.10 (CI 1.2–1.48)]. No literature exists concerning the risk of hypertension in men with a baseline BMI greater than 32 kg/m^2^.

We demonstrated that increasing WC values are generally associated with hypertension incidence after adjustment for confounders (especially BMI), irrespective of the baseline values of WC which is in line with the results of previous longitudinal population-based studies [20–22]. The Iranian National Committee of Obesity defined WC cut-offs of 90 cm in both genders as the cut-off point for screening for CVD risk factors including hypertension and diabetes [18]. In the current study, both the trajectories that showed higher risk of hypertension among women had a WC of 95 cm at baseline, however, the high increasing trajectory that had the greatest risk of hypertension showed a higher slope in WC. Similarly, among men, in the low-increasing group with normal BMI at baseline and higher slope in WC, showed significant risk for incident hypertension after controlling for BMI. In a representative population-based study among the Italian population, using two point measurements over about a decade, in a model adjusting for confounders, the risk of office, home, and 24-h hypertension for an increase of 1 cm of WC were 1.8%, 1.5% and 1.4%, respectively (all p values < 0.05) [23]. Moreover, among both Chinese men and women, those with or without abdominal obesity at baseline who had abdominal obesity at follow-up of 6 years showed significant risk for hypertension development independent of a large set of covariates (including baseline level of BMI) [22].

Increasing levels of WC apart from increasing insulin resistance can lead to hypertension through several well-known mechanisms [24]. Excessive fat tissues can contribute to sympathetic nervous system activation and secretion of molecules with the capacity of promoting the generation of aldosterone and angiotensin II, whose vasopressor and anti-natriuretic activities have been well-documented [24].

## TG trajectory and incident hypertension

We found similar trajectory patterns of TG trends for hypertension risk among both men and women. Approximately 90% of individuals of both genders stayed in the stable group over time. In the present study, only women with inverse U-shape or decreasing trajectories showed significant risk for incident hypertension in the fully adjusted model compared to the stable trajectory. Moreover, inverse U-shaped trajectories, comprising only about 3% of our population and having lower TG values at baseline compared to the decreasing trajectory (188 vs 298 mg/dl), showed significantly higher risk for incident hypertension in all three adjusted models. This phenomenon underlines not only the importance of the TG slope, but the superiority of the TG slope in predicting hypertension incidence compared to TG at intercept as well. Albeit several studies have affirmed that TG at baseline is a crucial factor for hypertension development [25, 26], no study, until now, has evaluated the association between hypertension incidence and TG trajectory.

In our study the association between TG trajectories and hypertension incidence among men disappeared after controlling for BMI, hence, it can be concluded that TG in men does not play an independent role in inducing hypertension; an issue that was previously found among TLGS participants using snapshot measurements of TG at baseline recruitment [27, 28]. The result of Zhang et al.’s study is similar to our results, highlighting the importance of TG in hypertension incidence especially among women [29]. However, an association between TG levels and incident hypertension in both sex groups independent of adiposity were shown in the Spanish population [30]. We previously demonstrated that during the last decade, obesity is showing an increasing trend, however, there is a decreasing trend for TG and TC concentrations among the Iranian population [35, 36]. This paradoxical situation is not restricted to the Iranian population and it was repeatedly shown in American as well as European populations, frequency of obesity is at an increasing pace whereas TG trend was downward in men and women [37–39]. Even in our data analysis among those with Inverse U-shape TG trajectory, when we examined changes of WC in this group, WC increased from 85 to 93 and 85 to 97 in women and men, respectively, opposite to the TG changes (Additional file 6: Figure S5).

Although explaining the mechanism behind rising BP in individuals with increasing levels of TG was not the goal of the present study, a number of studies have attempted to clarify this issue, nevertheless, the exact pathophysiology mechanism has yet to be determined. An unfavorable lipid profile disturbs endothelial function, which leads to a variety of complications including impairment in vasoregulation [31], decrease in the compliance of arteries, increase in arterial stiffness [32], and renal dysfunction [33].In addition to these, ample evidence in confirmation of the role of dyslipidemia (especially high levels of TG) in insulin resistance which maintains a mandatory role in the pathogenesis of hypertension, provide the most plausible explanation for the mechanism of hypertension incidence in individuals with high levels of TG [34]. Currently, as far as we know, no study has assessed the reason behind inter-sex differences with regards to the potential role of TG in inducing hypertension. However, Zhang et al. speculated that this may be because of differences in sex hormones [29].

## HDL-C, FPG, and TC trajectories and incident hypertension

In the present study, For HDL-C, the trajectory patterns in men and women were alike. Both trajectories (Low-increasing and High-increasing) had increasing pattern with different intercepts. Regarding FPG, similar to HDL-C, men and women had the same trajectory patterns. In both gender, the majority of our cohort (Stable trajectory, reference group) stayed stable at around 90 mg/dL. It is noteworthy that only one pattern (Stable trajectory) was detected for men and women of the present study. As far as we are aware, no study has evaluated the correlations between HDL-C, TC, and FPG trajectories and incident hypertension. We did not find any significant associations between increasing values of HDL-C and FPG and incident hypertension.

We acknowledge that our study had some strengths. First, to the best of our knowledge, this is the only study that assessed the association between sex-specific WC and TG trajectories and hypertension incidence. Second, the association between TG and hypertension incident cannot be fully reliable if the length of the follow-up is short [26], hence, owing to the long duration of follow-up in the current study, our results can be trusted. Finally, although the association between hypertension and BMI in individuals with BMI above 32 kg/m^2^ has not yet been examined [40], we found that in individuals with a mean BMI of 35.4 kg/m^2^, BMI has a strong association with hypertension incidence and has superiority over WC in predicting the risk of hypertension.

As a limitation, while we adjusted three models for a variety of covariates, there is still a possibility of residual confounding. Nutritional status and physical activity play an important role in evaluating hypertension risk. Unfortunately, we did not have data regarding nutritional status for TLGS participants at the baseline recruitment. Moreover, since the tools for assessing physical activity level changed between the two baseline recruitments, we did not consider this covariate in our data analysis [10]. Third, this study was conducted in the metropolitan city of Tehran, hence, our findings might not be generalizable to rural areas.

During more than a decade of follow-up, we found significant differences between genders in the association between trajectories of WC and TG with incident hypertension using LCGMM. Accordingly, after controlling for traditional risk factors and BMI among women, both a higher value of WC at the baseline and particularly a higher slope, were strongly associated with greater risk of hypertension. However, among men, this higher risk for increasing trajectories of WC was limited to men with normal BMI at baseline. Furthermore, only among women, a trajectory with fast increasing TG value, despite lower levels at baseline, was strongly associated with hypertension incidence.

## Supplementary Information

**Additional file 1: Fig. S1.**

**Additional file 2: ** .

**Additional file 3: Fig. S2.**

**Additional file 4: Fig. S3.**

**Additional file 5: Fig. S4.**

**Additional file 6: Fig. S5.**

## Data Availability

The datasets used and/or analyzed during the current study are available from the corresponding author on reasonable request.

## Abbreviations

WC: Waist circumference
TG: Triglyceride
BP: Blood pressure
SBP: Systolic blood pressure
DBP: Diastolic blood pressure
BMI: Body mass index
FPG: Fasting plasma glucose
FBS: Fasting blood glucose
TLGS: Tehran Lipid and Glucose Study
LCGMM: Latent Class Growth Mixture Modeling
