# Supplementary material for: Sex specific trajectories of central adiposity, lipid indices, and glucose level with incident hypertension: 12 years Follow-up in Tehran lipid and glucose study
Source: J Transl Med. 2021 Feb 23;19:84. doi: 10.1186/s12967-021-02749-x (PMC7903760; doi:10.1186/s12967-021-02749-x)
Supplement: Supplementary file 1 — Additional file 1: Fig. S1. [file 12967_2021_2749_MOESM1_ESM.pdf]

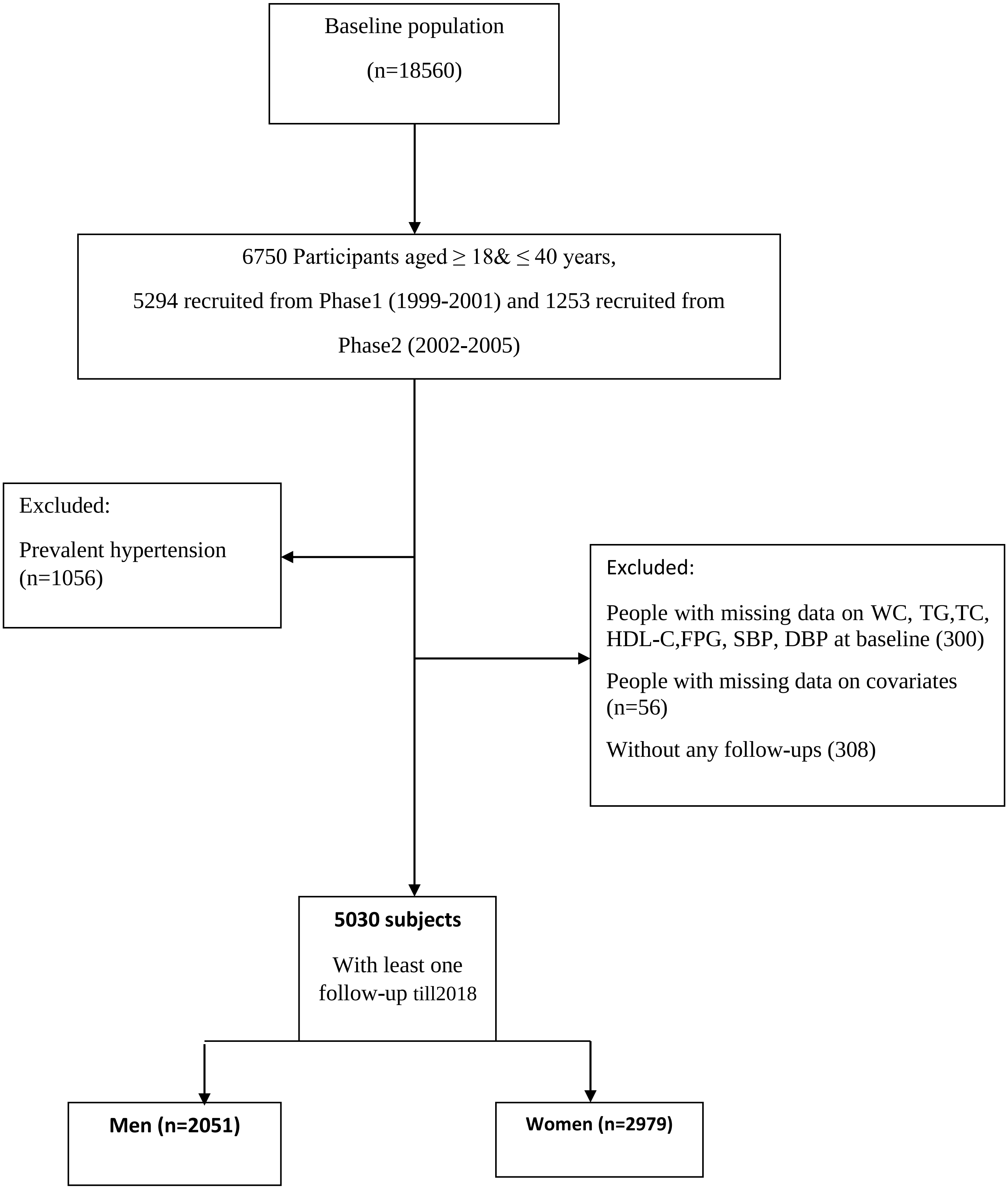

Supplementary Material Figure 1._ Flowchart of final analytical sample size for participants included in the study

TG: Triglyceride SBP: Systolic blood pressure DBP: diastolic blood pressure

WC: waist circumference TC: total cholesterol FPG : fasting plasma glucose
