# Supplementary material for: Sex specific trajectories of central adiposity, lipid indices, and glucose level with incident hypertension: 12 years Follow-up in Tehran lipid and glucose study
Source: J Transl Med. 2021 Feb 23;19:84. doi: 10.1186/s12967-021-02749-x (PMC7903760; doi:10.1186/s12967-021-02749-x)
Supplement: Supplementary file 2 — Additional file 2: . [file 12967_2021_2749_MOESM2_ESM.docx]

| Supplementary Material Table1. Model fit statistics for linear LCGMMof waist circumference for incident hypertension | | | | | | |
| --- | --- | --- | --- | --- | --- | --- |
| Number of classes | Number of parameters | AIC | BIC | ABIC | Entropy | A LMR test  P value |
| Females(n=2979) |  |  |  |  |  |  |
| Two | 14 | 90499.07 | 90579.06 | 90534.58 | 0.83 | 0.03 |
| Three* | 17 | 90452.94 | 90554.93 | 90500.91 | 0.84 | 0.07 |
| Four | 20 | 90428.13 | 90548.12 | 90484.57 | 0.79 | 0.11 |
| Five | 23 | 90414.59 | 90552.58 | 90479.50 | 0.75 | 0.41 |
| Six | 26 | 90401.84 | 90557.82 | 90475.2 | 0.74 | 0.22 |
|  |  |  |  |  |  |  |
| **Males(n=2051)** |  |  |  |  |  |  |
| Two | 14 | 58184.3 | 58263.1 | 58218.6 | 0.88 | 0.01 |
| Three* | 17 | 58151.8 | 58247.4 | 58160.8 | 0.90 | 0.06 |
| Four | 20 | 58140.7 | 58245.3 | 58181.7 | 0.89 | 0.08 |
| Five | 23 | 58123.1 | 58252.5 | 58179.4 | 0.68 | 0.18 |
| six | 26 | 58105.2 | 58251.3 | 58168.7 | 0.73 | 0.19 |
| LCGMM: latent class growth mixture modeling; AIC:Akaike information criterion; BIC: Information Criterion; ABIC: Adjusted Bayesian Information Criterion; A LMR test : Adjusted Lo-Mendell-Rubin likelihood ratio test  Lower BIC, AIC values indicate better fit. Higher entropy values indicate greater precision of class membership assignments. *The optimal class number according to the model fit criteria. | | | | | | |

| **Supplementary Material Table2.Model fit statistics for quadratic LCGMM of triglycerides for incident hypertension** | | | | | | |
| --- | --- | --- | --- | --- | --- | --- |
| Number of classes | Number of parameters | AIC | BIC | ABIC | Entropy | A LMR test  P value |
| **Females(n=2979)** |  |  |  |  |  |  |
| Two | 14 | 143182.59 | 143296.58 | 143236.21 | 0.94 | 0.04 |
| Three* | 17 | 142691.97 | 142829.95 | 142756.81 | 0.94 | 0.11 |
| Four | 20 | 12888.95 | 12999.03 | 12925.48 | 0.83 | 0.27 |
| Five | 23 | 12859.44 | 12993.38 | 12898.13 | 0.82 | 0.45 |
| Six | 26 | 12849.36 | 13001.16 | 12893.21 | 0.88 | 0.48 |
| **Males(n=2051)** |  |  |  |  |  |  |
| Two | 14 | 100936.18 | 101065.58 | 100992.50 | 0.96 | 0.04 |
| Three* | 17 | 101401.21 | 101508.112 | 101447.74 | 0.95 | 0.09 |
| Four | 20 | 100699.93 | 100851.835 | 100766.05 | 0.84 | 0.1 |
| Five | Not Identified |  |  |  |  |  |
| six | Not Identified |  |  |  |  |  |
| HPG: High plasma glucose; LCGMM: latent class growth mixture modeling; AIC:Akaike information criterion; BIC:Bayesian Information Criterion; ABIC: Adjusted Bayesian Information Criterion; A LMR test: Adjusted Lo-Mendell-Rubin likelihood ratio test. Lower BIC, AIC values indicate better fit. Higher entropy values indicate greater precision of class membership assignments. *The optimal class number according to themodel fit criteria. | | | | | | |

| **Supplementary Material Table 3. Baseline characteristics of study population by fasting plasma glucose trajectories for incident hypertension** | | | | | |
| --- | --- | --- | --- | --- | --- |
| **Females** | | | **stable**  **(n=2927)** | **increasing**  **(n=52)** | **P value** |
| Age (years) | | | 28.53(6.59) | 33.75(4.93) | 0.000 |
| Smoking status | | |  |  | 0.03 |
|  | (Current) (%) | | 0.6 | 1.9 | 0.6 |
|  | (paste) (%) | | 2.3 | 7.7 | 3.8 |
| BMI(kg/m2) | | | 25.15(4.6) | 30.20(4.55) |  |
| WC(cm) | | | 80.23(11.20) | 93.50(10.40) | 0.000 |
| Diabetes n (%) | | | 12(0.4) | 20(38.5) | 0.000 |
| TG( mg/dL ) | | | 97(73-135) | 128(168-233) | 0.000 |
| SBP (mmHg) | | | 107.24(10.27) | 112.32 (10.39) | 0.000 |
| DBP(mmHg) | | | 72.36(8.10) | 76.67(7.45) | 0.000 |
| BP, n (%) | | | 496 (16.9) | 19(36.5) | 0.000 |
| **Males** | | | **Stable**  **(n=1980)** | **Increasing**  **(n=71)** | **P value** |
| Age (years) | | | 29.04 (6.76) | 28.70 (6.34) | 0.000 |
| Smoking status | | |  |  | 0.2 |
|  | | (Current) (%) | 26.2 | 25.4 |  |
|  | | (paste) (%) | 9.1 | 24.5 |  |
| BMI(kg/m2) | | | 24.60(4.16) | 27.3(4.20) | 0.01 |
| WC(cm) | | | 84.11 (11.20) | 92.20(11.52) | 0.03 |
| Diabetes, n (%) | | | 15(0.8) | 13(18.3) | 0.04 |
| TG( mg/dL ) | | | 125(88-180) | 224(140-286) | 0.000 |
| SBP (mmHg) | | | 112.01 (10.44) | 114.77(10.7) | 0.000 |
| DBP (mmHg) | | | 73.80(8.10) | 77.55(7.88) | 0.000 |
| BP, n (%) | | | 462 (23.3) | 21(29.6) | 0.000 |
| Data are mean (standard deviation) for continues, and frequency (%) for categorical variables  BMI: Body mass index, DBP: Diastolic blood pressure, SBP: Systolic blood pressure; TG: Triglyceride | | | | | |

| **Supplementary Material Table 4**.Associations between fasting plasma glucose trajectories and incident hypertension | | | | | | | | | | |
| --- | --- | --- | --- | --- | --- | --- | --- | --- | --- | --- |
|  | | **Model1** | | | **Model2** | | | **Model3** | | |
|  |  | **Adjusted HR**  **(95% CI)** | **β** | **P**  **value** | **Adjusted HR**  **(95% CI)** | **β** | **P**  **value** | **Adjusted HR**  **(95% CI)** | **β** | **P**  **value** |
| **Females** | |  |  |  |  |  |  |  |  |  |
|  | Stable | Reference |  |  | Reference |  |  | Reference |  |  |
|  | Increasing | 1.48(0.93-2.35) | 0.39 | 0.095 | 1.17(0.74-1.89) | 0.17 | 0.473 | 1.13(0.71-1.82) | 0.13 | 0.585 |
| **Males** | |  |  |  |  |  |  |  |  |  |
|  | Stable | Reference |  |  | Reference |  |  | Reference |  |  |
|  | Increasing | 0.77(0.49-1.20) | -0.25 | 0.253 | 0.71(0.73-1.11) | -0.33 | 0.137 | 0.70(0.45-1.10) | -0.36 | 0.127 |
| **CI**: Confidence interval,  **HR** : Hazard ratio;  Model1: was adjusted for age, SBP, DBP  Model2: was adjusted for age, SBP, DBP, BMI  Model3: was adjusted for age, SBP, DBP, Smoking Status,, BMI, HDL-C | | | | | | | | | | |

| **Supplementary Material Table** 5. Baseline characteristics of study population by HDL-C trajectories for incident hypertension | | | | | |
| --- | --- | --- | --- | --- | --- |
| **Females** | | | **Low increasing**  **(n=2759)** | **High increasing**  **(n=220)** | **P value** |
| Age (years) | | | 28.62(6.61) | 28.70(4.60) | 0.11 |
| Smoking status | | |  |  | 0.03 |
|  | (Current) (%) | | 0.2 | 1.4 |  |
|  | (paste) (%) | | 0.4 | 2.7 |  |
| BMI(kg/m2) | | | 25.36(4.5) | 23.20(4.22) | 0.04 |
| WC(cm) | | | 80.10(11.34) | 76.40(10.20) | 0.000 |
| Diabetes n (%) | | | 32(1.2) | 0(00.0) | 0.000 |
| TG( mg/dL ) | | | 102(75-148) | 69(54-92) | 0.000 |
| SBP (mmHg) | | | 107.25(10.26) | 106.32 (10.09) | 0.07 |
| DBP(mmHg) | | | 72.51(8.11) | 71.67(7.20) | 0.06 |
| BP, n (%) | | | 485(17.6) | 29(13.2) | 0.000 |
| **Males** | | | **Low increasing**  **(n=1913)** | **High increasing**  **(n=138)** | **P value** |
| Age (years) | | | 29.00(6.36) | 28.7 (7.07) | 0.000 |
| Smoking status | | |  |  | 0.05 |
|  | | (Current) (%) | 26.4 | 22.5 |  |
|  | | (paste) (%) | 9.1 | 10.1 |  |
| BMI(kg/m2) | | | 24.98(4.10) | 23.3(4.21) | 0.01 |
| WC(cm) | | | 85.11 (11.23) | 81.17(11.4) | 0.03 |
| Diabetes, n (%) | | | 25(1.3) | 28(1.4) | 0.04 |
| TG( mg/dL ) | | | 138(93-220) | 85(63-124) | 0.04 |
| SBP (mmHg) | | | 111.36 (10.37) | 114.77(10.7) | 0.05 |
| DBP (mmHg) | | | 73.60(8.21) | 73.59(7.98) | 0.06 |
| BP, n (%) | | | 459 (24) | 24(17.4) | 0.000 |
| Data are mean (standard deviation) for continues, and frequency (%) for categorical variables  BMI: Body mass index, DBP: Diastolic blood pressure, SBP: Systolic blood pressure; TG: Triglyceride | | | | | |

| **Supplementary Material Table 6. Associations between HDL-C trajectories and Hypertension** | | | | | | | | | | |
| --- | --- | --- | --- | --- | --- | --- | --- | --- | --- | --- |
|  | | **Model1** | | | **Model2** | | | **Model3** | | |
|  |  | **Adjusted HR**  **(95% CI)** | **β** | **P**  **value** | **Adjusted HR**  **(95% CI)** | **β** | **P**  **value** | **Adjusted HR**  **(95% CI)** | **β** | **P**  **value** |
| **Females** | |  |  |  |  |  |  |  |  |  |
|  | Low -increasing | Reference |  |  | Reference |  |  | Reference |  |  |
|  | High- increasing | 0.71(0.49-1.03) | -0.33 | 0.078 | 0.78(0.53-1.14) | -0.25 | 0.206 | 0.78(0.53-1.19) | -0.24 | 0.206 |
| **Males** | |  |  |  |  |  |  |  |  |  |
|  | Low -increasing | Reference |  |  | **Reference** |  |  | **Reference** |  |  |
|  | High- increasing | 0.71(0.47-1.08) | -0.33 | 0.113 | 0.77(0.51-1.17) | -0.24 | 0.238 | 0.76(0.50-1.16) | -0.26 | 0.218 |
| **CI**: Confidence interval,  **HR** : Hazard ratio;  Model1was adjusted for age, SBP, DBP  Model2was adjusted for age, SBP, DBP, BMI  Model3: was adjusted for age, SBP, DBP, Smoking Status, BMI, HDL-C | | | | | | | | | | |

| **Supplementary Material Table 7. Baseline characteristics of study population by total cholesterol trajectories for incident hypertension** | |
| --- | --- |
| **Females** | **stable**  **(n=2979)** |
| Age (years) | 28.62(6.61) |
| Smoking status |  |
| (Current) (%)  (paste) (%) | 0.2 |
|  | 0.4 |
| BMI(kg/m2) | 25.36(4.5) |
| WC(cm) | 80.10(11.34) |
| Diabetes n (%) | 32(1.2) |
| TG( mg/dL ) | 102(75-148) |
| SBP (mmHg) | 107.25(10.26) |
| DBP(mmHg) | 72.51(8.11) |
| BP, n (%) | 514 (17) |
| **Males** | **Stable**  **(n=2051)** |
| Age (years) | 29.00(6.36) |
| Smoking status |  |
| (Current) (%)  (paste) (%) | 26.4 |
|  | 9.1 |
| BMI(kg/m2) | 24.98(4.10) |
| WC(cm) | 85.11 (11.23) |
| Diabetes, n (%) | 25(1.3) |
| TG( mg/dL ) | 138(93-220) |
| SBP (mmHg) | 111.36 (10.37) |
| DBP (mmHg) | 73.60(8.21) |
| BP, n (%) | 483 (23) |
| Data are mean (standard deviation) for continues, and frequency (%) for categorical variables BMI: Body mass index, DBP: Diastolic blood pressure, SBP: Systolic blood pressure; TG: Triglyceride | |

| **Supplementary Material Table 8.** Associations between waist circumference trajectories and hypertension | | | | | | | |
| --- | --- | --- | --- | --- | --- | --- | --- |
| **Males** | | **Adjusted HR**  **(95% CI)** | **P**  **value** | **Adjusted HR**  **(95% CI)** | **P**  **value** | **Adjusted HR**  **(95% CI)** | **P**  **value** |
|  | Low –increasing | Reference |  | Reference |  | Reference |  |
|  | Stable | 0.38(0.21-0.71) | 0.00 | 0.63(0.32-1.23) | 0.17 | 0.64(0.33-124) | 0.20 |
|  | High- increasing | 0.90(0.39-2.07) | 0.82 | 1.66(0.67-4.12) | 0.26 | 1.61(0.64-4.12) | 0.30 |
| **CI**: Confidence interval,  **HR** : Hazard ratio;  Model1was adjusted for age, SBP, DBP  Model2was adjusted for age, SBP, DBP, BMI  Model3: was adjusted for age, SBP, DBP, Smoking Status, Diabetes, BMI, HDL-C | | | | | | | |
