# Supplementary material for: Sex specific trajectories of central adiposity, lipid indices, and glucose level with incident hypertension: 12 years Follow-up in Tehran lipid and glucose study
Source: J Transl Med. 2021 Feb 23;19:84. doi: 10.1186/s12967-021-02749-x (PMC7903760; doi:10.1186/s12967-021-02749-x)
Supplement: Supplementary file 3 — Additional file 3: Fig. S2. [file 12967_2021_2749_MOESM3_ESM.pdf]

a

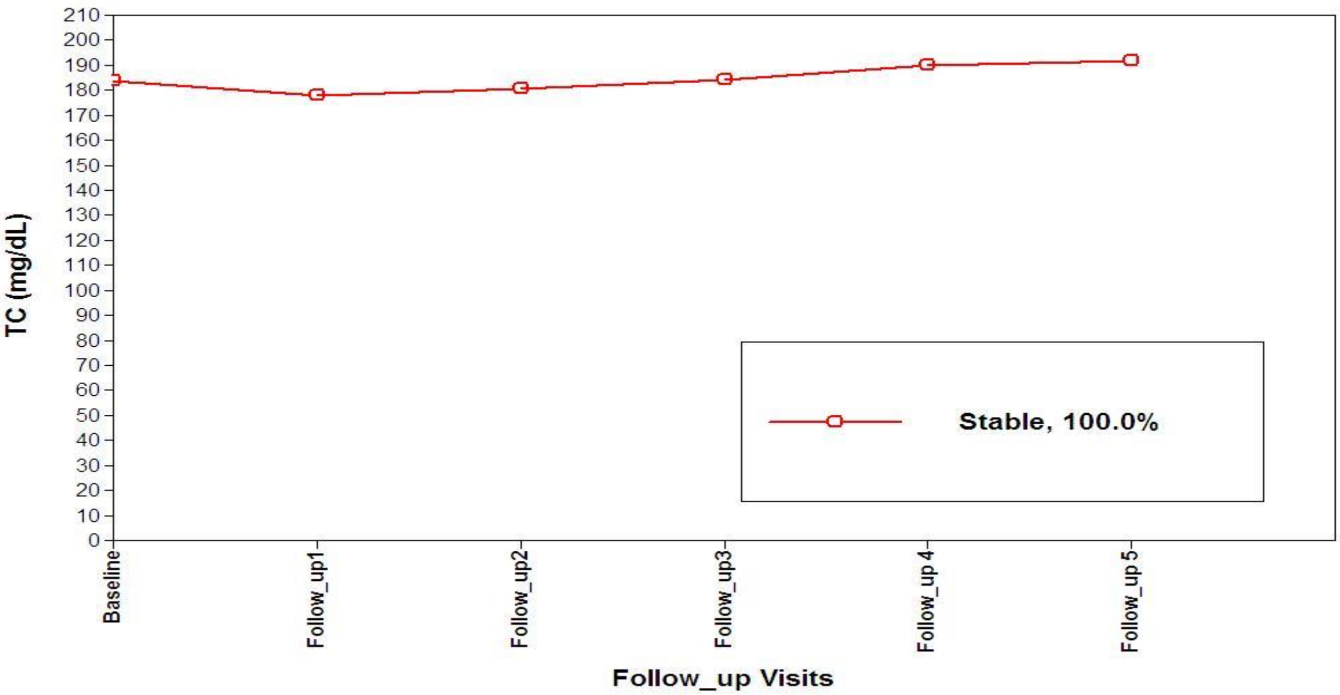

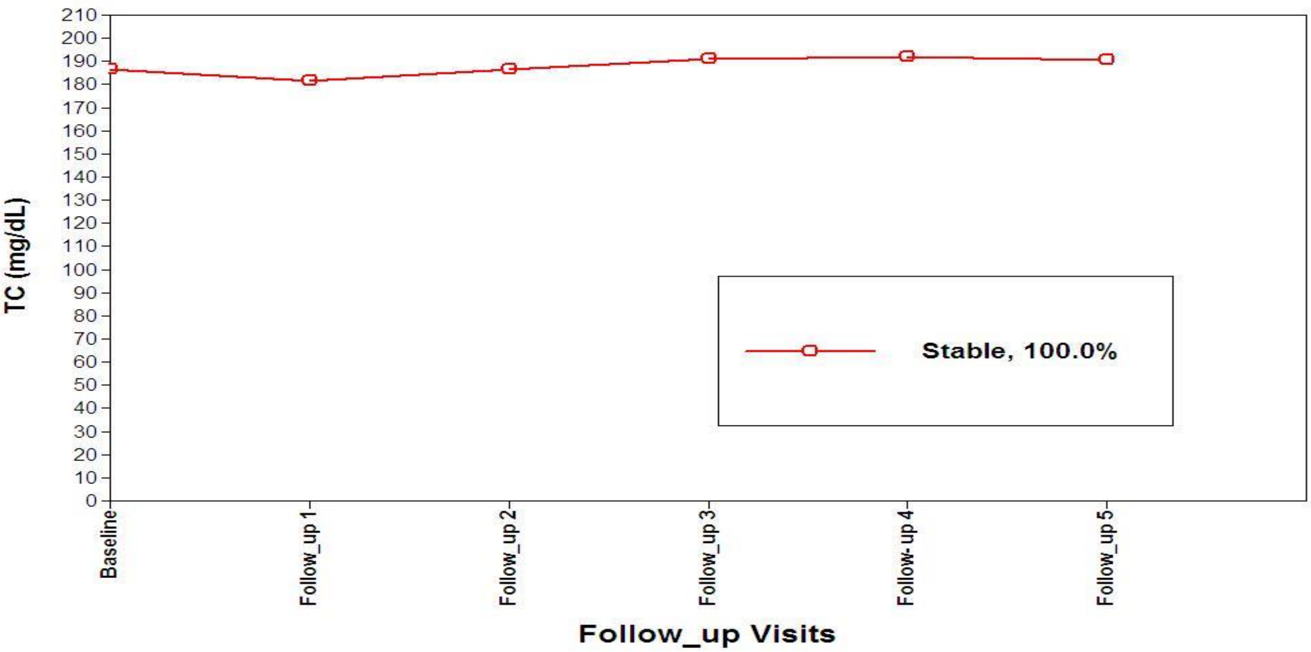

b

Supplementary Material _ Figure2. linear latent trajectory classes of TC among women for incident hypertension. **b** latent trajectory classes of TC among men for incident hypertension
