# Supplementary material for: Sex specific trajectories of central adiposity, lipid indices, and glucose level with incident hypertension: 12 years Follow-up in Tehran lipid and glucose study
Source: J Transl Med. 2021 Feb 23;19:84. doi: 10.1186/s12967-021-02749-x (PMC7903760; doi:10.1186/s12967-021-02749-x)
Supplement: Supplementary file 4 — Additional file 4: Fig. S3. [file 12967_2021_2749_MOESM4_ESM.pdf]

a

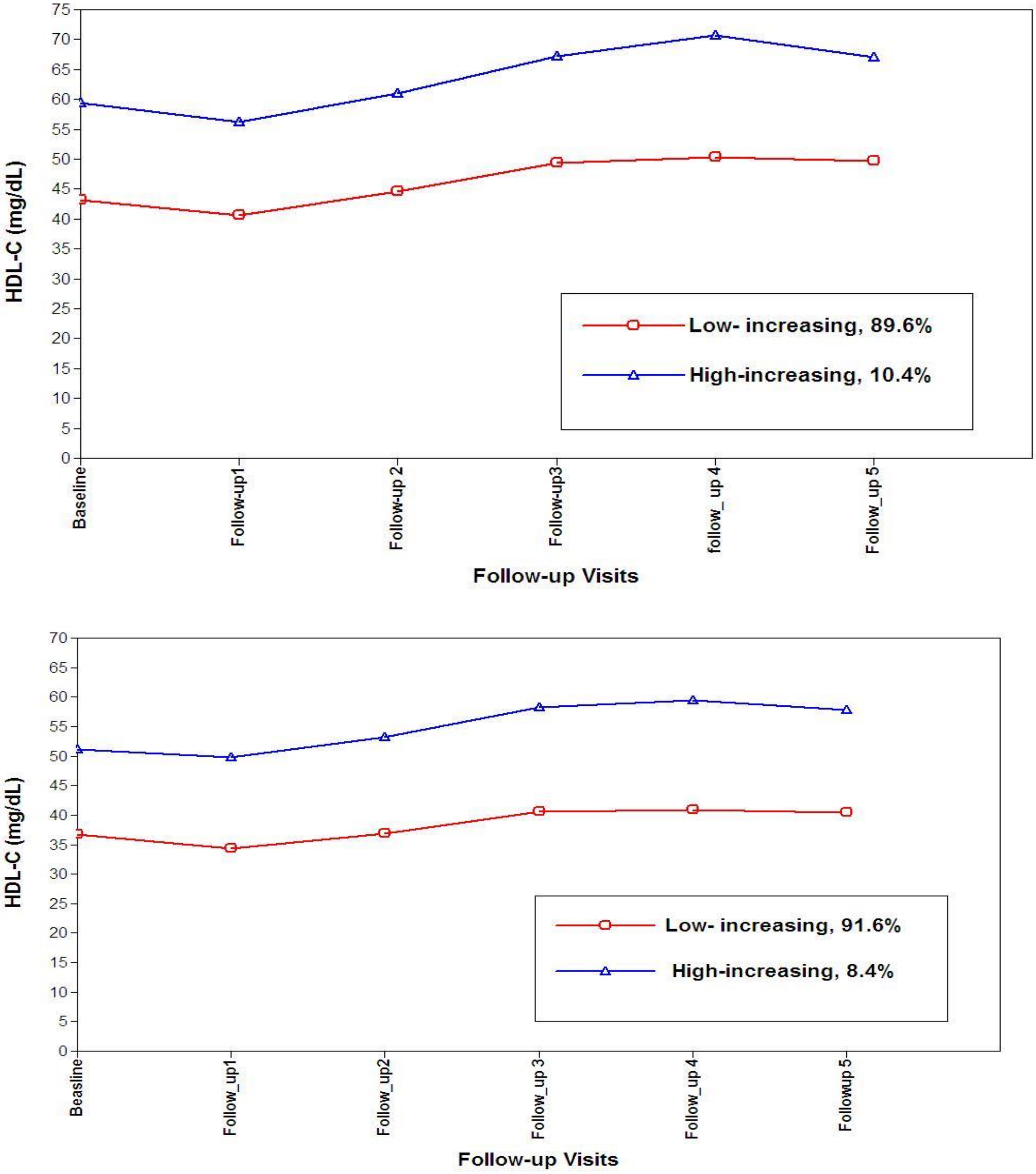

b

Supplementary Material _ Figure3. linear latent trajectory classes of HDL-C among women for incident hypertension. **b** latent trajectory classes of HDL-C among men for incident hypertension
