# Supplementary material for: Sex specific trajectories of central adiposity, lipid indices, and glucose level with incident hypertension: 12 years Follow-up in Tehran lipid and glucose study
Source: J Transl Med. 2021 Feb 23;19:84. doi: 10.1186/s12967-021-02749-x (PMC7903760; doi:10.1186/s12967-021-02749-x)
Supplement: Supplementary file 6 — Additional file 6: Fig. S5. [file 12967_2021_2749_MOESM6_ESM.pdf]

**a**

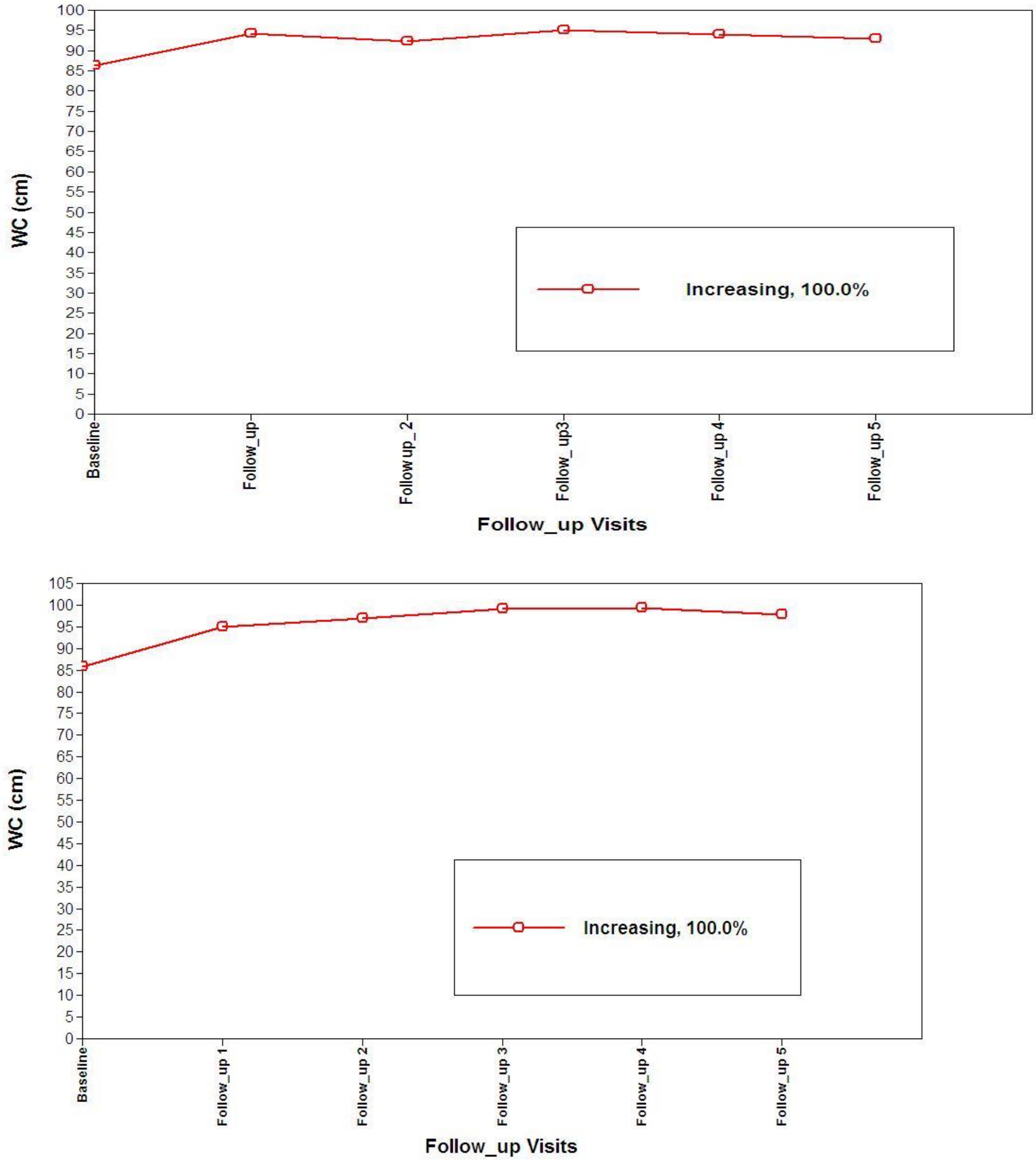


**Supplementary Material _ Figure5.** Change of WC among women in **Inverse U- shape trajectory** for incident hypertension. **b** Change of WC among men in **Inverse U- shape trajectory** for incident hypertension
